# Self-managed programmes in homeless care as (reinvented) institutions

**DOI:** 10.1080/17482631.2020.1719002

**Published:** 2020-01-24

**Authors:** Max A. Huber, Rosalie N. Metze, Martin Stam, Tine Van Regenmortel, Tineke A. Abma

**Affiliations:** a Research Center for Societal Innovation, Amsterdam University of Applied Sciences, Amsterdam, The Netherlands; b Academic Collaborative Center for Social Work, Tilburg University, Tilburg, The Netherlands; c HIVA Research Institute for Work and Society, Catholic University Leuven, Leuven, Belgium; d Department of Medical Humanities, Free University Amsterdam, Amsterdam, The Netherlands

**Keywords:** Self-managed programme, institutionalization, freedom of choice, homeless care, social work, peer work

## Abstract

**Purpose**: Self-managed institutional homeless programmes started as an alternative to regular shelters. Using institutional theory as a lens, we aim to explore the experiences of stakeholders with the institutional aspects of a self-managed programs.

**Method**: The data we analysed (56 interviews, both open and semi-structured) were generated in a longitudinal participatory case-study into JES, a self-managed homeless shelter. In our analysis we went back and forth between our empirical data and theory, using a combination of systematic coding and interpretation. Participants were involved in all stages of the research.

**Results**: Our analysis revealed similarities between JES and regular shelters, stemming from institutional similarities. Participants shared space and facilities with sixteen people, which caused an ongoing discussion on (enforcement of) rules. Participants loathed lack of private space. However, participants experienced freedom of choice over both their own life and management of JES and structures were experienced more fluid than in regular care. Some structures also appeared stimulated self-management.

**Conclusion**: Our analysis showed how an institutional context influences self-management and suggested opportunities for introducing freedom and fluidity in institutional care.

In self–managed institutional homeless care, participants and their peers are responsible for both day to day affairs and strategic decisions, such as whether to move. Professionals give advice, they have no formal say (Tuynman & Huber, 2014). Self-managed homeless care is a form of self-organized care, which is associated with individual and collective empowerment (Brown, 2012). An institutional context appears to influence self-management (Huber et al., n.d.), in line with the influence of the institutional context in regular institutional care (Abma, 2010; Enarsson, Sandman, & Hellzén, 2008; Wolins & Wozner, 1982). Goffman described care institutions or *total institutions* as: “a place [… .] where a large number of like-situated individuals, cut off from the wider society for an appreciable period of time, together lead an enclosed, formally administered [… .] life” (1961:xiii). The dominant policy in the Netherlands and other Western countries is to stimulate deinstitutionalization of people living in clinics, shelters or sheltered living facilities, however, many former institutionalized residents struggle to sustainably do so (Kroon, 2018). There is increasing attention for supporting recovery within institutional care, though organizations struggle to put this to practice (Kroon, 2018; Slade et al., 2014).

Using insights from institutional theory and research on institutional care (Goffman, 1961; Scott, 2010; Wolins & Wozner, 1982) we aim to understand the influence of an institutional context on self-management. Institutional theory describes how social structures, such as rules, norms and routines, influence social behaviour and how social behaviour in turn influences social structures (Scott, 2005). Little to no attention is given in literature on self-organized care to institutional influences (Brown, 2012). We aim to further our insight into the meaning of self-managed institutional programs compared to regular programs from the perspectives of multiple stakeholders, including participants, peer workers and social workers. We expect that using self-managed programs as an outlier case (Gerring, 2007) will offer new insights into regular institutional care.

## Total institutions and institutional theory

Goffman describes total institutions as “social hybrids, part institutional community, part formal organization” (1961, p. 12), organizing every part of daily life. There is a strong focus on maintaining order among large groups of residents (Goffman calls them *inmates* in all contexts), with little staff and limited resources. Total institutions are “staging a difference between two constructed categories of persons” (1961, p. 111), forcing a *binary division*, you are either staff or inmate (1961, p. 7), enforced by different dress codes and different required behaviour. During *role releases*, the social distance between staff and residents becomes smaller and residents have more freedom, but they are also expected to behave better (Goffman, 1961). Compliance from residents is sought from the entrance, where residents have to hand in personal belongings, *stripping identity* (Goffman, 1961). Staff has more information than residents, both in general and concerning residents, which stimulates compliance. Reduction of an individual to a sole role of inmate, *mortification* (1961, p. 21), harms the self-image of residents and stimulates adaptation to the workings of the institution, to the extent that it hinders the ability to function outside the institution (Goffman, 1961).

Residents live in *batches*: they eat, recreate and sleep together, at regulated times. Individual needs or desires cost more time and make it harder to maintain order for staff. Rules, privileges and withdrawal of privileges are used to stimulate compliance. Residents with different levels of privileges may harm each other’s privileges. Having more people within a programme increases the risk that residents with varying degrees of privilege live together, which in turn increases the need for control and limits customized care (Goffman, 1961). In shelters, authors refer to *shelterization* (Grunberg & Eagle, 1990; Keigher, 1992; Stark, 1994). Either out of efficiency (Keigher, 1992), pessimism and cynicism (Grunberg & Eagle, 1990) or safety (Stark, 1994), it is argued that staff in shelters focus on rules, routines and regulations, through privileges, punishment and a prohibition on disturbing efficiency (Grunberg & Eagle, 1990; Keigher, 1992; Stark, 1994). Institutionalization can be stimulated by external bureaucratic demands, such as financial changes, enforced protocols or required accountability (Cain, 2019; Goffman, 1961; Wolins & Wozner, 1982). Physical aspects of an institution can strengthen negative influences, for instance through a lack of private space (Goffman, 1961).

Some argue that all institutional care settings are essentially similar, dealing with similar issues (Wolins & Wozner, 1982) and focused on two main tasks: developing skills (voluntary or forced) and offering a place to stay for residents who want to, are forced to or who are not able to stay anywhere else (Wozner, 1990). Which task is dominant differs, depending on several aspects of the setup: broad to specific target group; complete to no care; voluntary to forced stay; short to permanent stay and as a consequence of the latter: varying or stable population (Wolins & Wozner, 1982; Wozner, 1990). The process of institutionalization in an institutional setting has been described in closed programs such as prisons (Wolins & Wozner, 1982), homes for older people (Abma, 2010) and youth (Hanrath, 2013) and even tourist locations (e.g., resorts, cruise ships, amusement parks), because, although voluntary and short term, the period that tourists spend there, is highly regulated (Ritzer & Liska, 2004). In this paper we define institutional programs as physical places were unnatural groups stay in a (more or less) structured fashion for a similar purpose: to develop skills and/or a place to stay (Goffman, 1961; Wolins & Wozner, 1982).

Recent institutional theory argues that the influence between institutions and individuals is bidirectional (Scott, 2005). Individuals have an influence by conforming to, reproducing or rejecting a structure (DiMaggio & Powell, 1983; Giddens, 1984). Especially when roles are unclear, changing or conflicted, (creating) structure can offer control and certainty (DiMaggio & Powell, 1983; Giddens, 1984; Kruiter, De Jong, Van Niel, & Hijzen, 2008). Stress, ambiguity and insecurity are inherent in the work of staff in institutional care, they are simultaneously responsible for individuals, a group and order while their clients combine complex personal problems with negative coping strategies and negative experiences with care (Enarsson et al., 2008; Goffman, 1961; Keigher, 1992). Staff and clients can get stuck in vicious circles of distrust; negative behaviour results in rule enforcement, leading to more negative behaviour and a growing divide between staff and residents (Goffman, 1961; Van Der Helm & Schaftenaar, 2014; Wolins & Wozner, 1982). Hanrath (2013) describes how staff and residents both try to gain and maintain control by interpreting each other’s behaviour and anticipate interpretation.

Organizational interventions can help break through institutionalized behaviour, such as enforcing consumer rights, offering role certainty and predictability for both clients and staff, creating transparency and offering opportunities for participation (Hoijtink & Oude Vrielink, 2007; Kruiter et al., 2008; Schön, Grim, Wallin, Rosenberg, & Svedberg, 2018; Scott, 2005). It is argued that increased space for participation of both clients and staff, more custom made care and higher staff satisfaction is associated with better outcomes for clients (Jongepier, Struijk, & Van Der Helm., 2010; Schön et al., 2018). Staff satisfaction is stimulated by institutional and practical support (Chou & Robert, 2008). Structure can help staff to maintain a feeling of control in their daily existence and the insecurities they face (DiMaggio & Powell, 1983; Giddens, 1984; Kruiter et al., 2008).

Therapeutic communities, like self-managed programs, started as an alternative to regular institutional programs, using living together as treatment, although critics say therapeutic communities are a subtle form of social control (Bloor, 1986). Scott (2010) calls alternative forms of support, such as self-help programs, *reinvented institutions*, because they still aim to stimulate identity transformation. Rituals in reinvented institutions are subtle forms of self- and social control, according to Scott (2010), for example when participants press each other to conform to house rules. Staff is no longer needed, because participants have internalized self and social control (Scott, 2010).

In the Netherlands, where we did our research, the first two self-managed programs started in the nineties, building on earlier experiences with alternative institutional programs (Tuynman & Huber, 2014). Together with squatter groups, homeless people themselves started self-managed homeless shelters as a protest against a lack of place in shelters and a perceived paternalistic approach in regular shelters. Their claim was (and is) that people who are or have been homeless themselves, are better able to run a homeless shelter than professionals in regular homeless care organizations. These grassroots level programs initiated by people themselves still exist, although both became part of a regular homeless care organization. New self-managed programs are most often initiated by or together with regular care organizations. In the first and the second decennium of this century, many new programs were started in the Netherlands, although some already ended as well (Tuynman & Huber, 2014).

Self-managed shelters reach people who are not (yet) able to access housing. Participants and peer workers are in charge of daily and strategic affairs in self-managed shelters (Tuynman & Huber, 2014). Relatively little research has been done into self-managed shelters (Tuynman & Huber, 2014). More is known about other self-organized programs, from consumer-run centres (Brown, 2012) to peer-run respite houses (Ostrow & Croft, 2015). Self-organized programs are managed by participants and peer workers and emphasize empowerment (Brown, 2012; Ostrow & Croft, 2015). Those who initiated self-managed shelters claim that these settings are an alternative to regular shelters, in offering more freedom to participants. Although research on self-managed shelters is lacking, research into other self-organized programs suggests that they are associated with psychological empowerment (Brown, 2012).

Self-managed institutional programs in homeless care started as an alternative to regular institutional care, while at the same time sharing institutional characteristics. In this paper we aim to explore the experiences of stakeholders with the institutional aspects of a self-managed programs by answering the following research questions: To what extent do stakeholders experience a self-managed shelter as an alternative for regular shelters? Which similarities and which differences are experienced by stakeholders between a self-managed shelter and a regular shelters? And how do stakeholders experience processes of structuration within a self-managed shelter?

## Method

The empirical data for this paper stems from a longitudinal participatory case study (2009–2016) (Abma & Stake, 2014) of empowerment processes of participants in Je Eigen Stek [Your Own Place, JES], a self-managed shelter, that started in 2008 in the Netherlands. The research is part of the Collaborative Centre for the Social Domain (Werkplaats sociaal domein) at the Amsterdam University of Applied Sciences.

Using a case study methodology does justice to the complex nature of a self-managed shelter and fits with the social-constructivist approach of our research, aimed at understanding the unique experiences of participants and other stakeholders (Abma & Stake, 2014; Hyett, Kenny, & Dickson-Swift, 2014). A social-constructivist approach is also fitting with research into empowerment process (Van Regenmortel, 2011). In line with our social-constructivist approach, we followed the principles of *responsive evaluation* (Abma, Leyerzapf, & Landeweer, 2017; Abma, Nierse, & Widdershoven, 2009), where stakeholders are engaged in the process of evaluation (Abma, 2019). Issues of concern of stakeholders in relation to the meaning of self-management form the starting point for a dialogue to develop mutual understanding, articulate different perspectives and determine the merit of practices to improve quality of the evaluation. The evaluation has been executed by a diverse team of researchers, including researchers with lived experience with homelessness. Participants, peer workers and social workers from JES engaged in co-designing the research, developing topic-lists, recruiting respondents, co-interviewing, discussing the outcomes of analysis and contributing to publications.

### Study setting

JES serves people who are homeless and are not (yet) able to obtain independent housing, because of financial and personal problems and/or issues in accessing housing because of a shortage in social housing (Padgett, Henwood, & Tsemberis, 2016; Tuynman & Planije, 2014; Van Straaten et al., 2016). A specific motivation of participants for starting and joining JES was a dissatisfaction with perceived paternalism and fragmentation in regular shelters.

JES has room for sixteen people, mostly men, who want to work on their own problems in their own way and are able to take care of themselves, according to themselves and other participants. The stated goal of JES is “to help people without a home, get a home.” Most participants are dependent on welfare, some have a job. The participants are responsible for the management, from household to entrance and exit of participants and strategic issues. JES is funded by the municipality of Amsterdam and is part of a larger organization which offers regular homeless care. JES has hired a social worker to support individual participants, the group and the program, besides the social worker that facilitated the development of JES and is involved at a greater distance. The social worker collaborates with a peer worker, a former participant, both are paid.

In the first five years of JES (2009–2014), 72 people joined, from less than a day to multiple years. If we exclude those who leave (almost) immediately (stay less than three months), the average length of stay is around fifteen months. Of the 72 participants, 51 stayed for more than three months, of whom 32 were explicitly spoken to as part of our research (interview or informal meeting), from seventeen others we have secondary information (from informal meetings, key informants and administrative data), such as next place of stay and reason for leaving (e.g., conflict, debt, found alternative place to stay).

### Data collection

Our formal data consists of interviews with participants (N = 27), peer workers (N = 3), social workers (N = 2) and other stakeholders (N = 10), the latter were either policy advisors from the mother organization of which JES is part, who supported the development of JES, or representatives from partner organizations such as housing organizations, the municipality and local social work organizations). Some of the participants, peer workers and social workers have been interviewed multiple times, resulting in 56 interviews. Eight participants were interviewed during their stay at JES, most were interviewed afterwards, varying from several weeks to several years after they left JES.

The interviews come from two sub-projects. The first was a case study into JES (2009–2010), for which open interviews (Bryman, 2008) were held. Interview questions aimed at understanding the perspective of stakeholders. Questions were among others: What is the current purpose of JES according to you? What are causes for some participants to participate more than others according to you?

The second sub-project (2013–2014) focused on how former JES-participants looked back at their participation using a semi-structured topic-list (Bryman, 2008), and how their life developed on several life domains (e.g., housing, finances, social contacts, day activities). Questions included: How did you spend your day during your stay at JES? How would you describe your interaction with other participants?

Interviews for the first study have been done by two academic researchers, one of whom is the first author. Interviews from the study into former participants have been done by couples of participants and students, under the supervision of experienced researchers, one of whom is the first author. All interviews, both from the first and second study, were recorded and transcribed. In addition to the interviews, documents delivered by respondents to the first author were analysed (e.g., documents containing current and future developments of the program and auto-publications by participants).

From the start of the first study up until the present, the first author engaged with participants, peer workers and social workers from JES, based on an ethnographic and participatory approach (O’Reilly, 2012), to developing long lasting relations, from 2009 to the present. The prolonged engagement and persistent observations (Lincoln & Guba, 1985) allowed the first author to gain a deeper understanding of the interview data and to observe changes overtime and the interaction between participants, peer workers and social workers among themselves and with outsiders (including the researchers). The interactions and observations done in this time focused on gaining more insight into how participants and other stakeholders experienced self-management over time. Because they were not collected as (structured) observational data, they have not been used for the primary analysis.

### Analysis

In our analysis we went back and forth between our empirical data and the theory, using a combination of interpretation and systematic coding, assisted by MAXqda. To manage our large dataset, we started by creating thematic categories. We developed working hypotheses to guide our focus, based on both an open coding of the empirical data by different researchers, among who the first author, and different theoretical concepts (O’Reilly, 2012). The analysis presented in this paper is part of a broader analysis of empowerment processes within JES. During the broader analysis we recognized that JES was less of a radical alternative to regular programs than suggested by the initial instigators and proponents of self-management, which we decided needed specific analysis. Themes that emerged out of the data included a focus on rules and procedures, a lack of private space and respondents explicitly comparing aspects of JES with regular shelters. Inspired by the “plugging in” approach of Jackson and Mazzei (2013), we explored core themes in literature on institutional care and institutional theory to increase our understanding of the data. Themes that we used from the literature include mortification, influence of actors on institutionalization, fluidity vs. rigidity of structures and the role of space. Building on the themes defined out the data and the literature, a code-tree was developed and refined through axial coding, starting with open coding within a theme, defining and adapting subthemes as we went along, and then going back to refine earlier coding. Our analysis was neither deductive nor inductive, rather it was iterative, that is a back-and-forth movement between data and interpretations, using the institutional theory as a lens for understanding the data (O’Reilly, 2012). The analysis has been executed by the first author, under supervision of the other authors. The final code tree has been tested by the second author. Main themes on the code tree are: mimicry of regular programs; fluidity vs institutionalism; influence of institutional setting on empowerment; setup of the program; management of the program; actors. Through the different phases in and approaches to our analysis, we have developed a *thick analysis* (van Staa & Evers, 2010).

The richness of perspectives and the different theoretical approaches, allowed us to make room for competing explanations (Abma et al., 2009). We strived for an authentic and recognized representation of the different perspectives involved with JES (Abma & Stake, 2014; Lincoln & Guba, 1985), paying explicit attention to the risk of overrepresentation of more reflexive respondents (Bryman, 2008). We used several forms of triangulation: different types of data gathering, different researchers and different analytical approaches to limit the risk of bias (Denzin, 1989). Through triangulation, a transparent method description and describing our rationale for selecting this case, we aimed to improve the quality our case study (Hyett et al., 2014).

Throughout the analysis we have remained in contact with JES, discussing preliminary analyses and working hypotheses with participants, peer workers and social workers in multiple sessions. This sharpened the analysis and increased the authenticity and a shared understanding of the core findings (Doyle, 2007; Lincoln & Guba, 1985). For both studies a draft version of a report was discussed with respondents and other stakeholders in focus groups. Member checks were performed at various stages: both preliminary findings, working hypotheses and draft version of conclusions were discussed with both respondents and other participants, peer workers and social workers involved and their input has been processed. This is in line with Lincoln and Guba (1985) who see member checking as a process that occurs continuously during the research project, both informal and formal, and comprises the testing of data, analytic categories, interpretations and conclusions with members of the stakeholder group(s). Agreement of the respondent group establishes the credibility of the researchers work and is a “strong beachhead toward convincing readers and critics of the authenticity of the work” (Lincoln & Guba, 1985, p. 315). Member checking fits with our participatory evaluation approach (Abma, 2019). The datasets generated during the current study are not publicly available due to confidentiality issues, pertaining to the qualitative and personal nature of the interviews. Datasets are available from the corresponding author on reasonable request.

### Ethical considerations

In our research we have complied with APA ethical principles in the treatment of individuals. Executives of participating organizations assessed the legal and ethical implications of the study, and approved the procedures. Our research meets the requirements of anonymity, consent, confidentially and safety of the participants and was guided by the ethical principles autonomy, beneficence, non-maleficence, and justice. Participants were verbally informed on the purpose of the research and our use of their information. Written consent at one point in time fits less well with participatory research into marginalized groups (Abma et al., 2019; Miller & Bell, 2002). In providing consent, respondents were given the option to withdraw their consent at any time, which was done by one participant, whose interviews were deleted.

## Findings

Our findings sections consists of four parts, following the stages in our analysis. Firstly, we explore to what extent JES is an alternative to regular programs from the perspectives of multiple stakeholders, outlining similarities and differences that are further explored in the second and third part. Following themes described in the literature and emerging in our data, we compare similarities and differences between JES and regular programs. Finally, inspired by recent institutional theory on structuration, we explore how structuration took place in JES. The cited quotes of respondents are translated from Dutch by the first author.

### JES as an alternative to regular programs

JES was started for participants who wanted more freedom than was offered in regular programs. To our surprise it emerged in our data that JES was less of a radical alternative than the initiators originally expected. In this first part we explore the lived experiences and to what extent the stakeholders experienced JES as an alternative to regular programs.

Participants loathed regular programs for an abundance of rules, unwanted interference from staff and a lack of acknowledgement of their capacities.

“You had more freedom than in regular care [in JES][…][In regular homeless care] they are constantly watching you, to see if they can tell you off, and you are obligated to get up at a certain time, and you have to be back at a certain time” (participant).

“In regular shelters, everyone gets the same standard package of care. Everyone is treated as a baby” (participant/peer-worker).

In JES there was a strong emphasis on freedom of participants. Participants experienced self-management in different ways. On one end of the spectrum, participants experienced JES as very empowering.

“It was a delight. You get your own keys, you can enter when you want. You can have input to everything concerning JES, in project groups, during meeting. Yes that was nice, that you could contribute“ (participant).

On the other end of the spectrum participants were negative, sharing disappointment about JES, both in relation to other participants and to a lack of freedom. In between those two ends of the spectrum were participants who were not engaged with social processes and the management of JES, although they enjoyed the freedom JES offered to work out their problems in their own way. The same freedom led to some participants getting stuck in what participants call “the fyke of self-management” (the trap of self-management), they adapted to live within JES, without making progress, similar to the process of hospitalization.

“There were people, who did nothing, truly nothing, to improve their situation. They didn’t want to move on. They resigned themselves. They had food, they could sleep, could watch television and it cost them almost nothing” (participant).

All participants, even those who were in general positive, (also) described negative aspects of self-management, often in relation to having to live and manage together. Many of the experienced negative aspects mimicked the described critique of regular institutional care, such as a focus on rules, lack of acknowledgement of capacities and interference from others (albeit peers rather than staff).

“The new group [of participants] got no chance [… .] They behaved like in all shelters; there was no ownership, it didn’t feel like they had any influence [… .] so they did nothing” (Coach).

The mimicry was a surprise to us and to some of the participants.

### Similarities between JES and regular institutional programs

Although a surprise at first sight, further exploration of the data, in interaction with themes stemming from literature, revealed several similarities with regular programs such as the participants living together within a shared space, being part of a formal organization and the development of social distance between participants.

Both JES and regular programs consist of a physical space, where people live together in an unnatural group. Participants had personal problems, both pre-existing, caused by being homeless or as a consequence of mortification in previous care. Many participants described forms of learned helplessness and a general distrust towards others, either about themselves or others.

“You have to have a thick skin, because the people that are here, they are all homeless for a reason. [… .] They have a past, of which, sometimes, they are not proud, so they are suspicious [… .] waiting how the wind blows. They try to go for their own benefit” (participant).

JES is relatively small compared to regular shelters that in the Netherlands host up to 60 people. Nevertheless, participants mentioned that living “with sixteen men with backpacks full of pain and sorrow [… .] all those emotions, at a certain point, is bound to collide” (participant). Participants of JES lived in a shared building, with shared facilities, a shared living room and often a shared bedroom, which caused tensions. Participants varied though in both what they experienced as negative about living together and to what extent they experienced it as negative. Some participants complained that they were forced to do the dishes, others complained that they had to reproach fellow participants about doing the dishes. Similarly, some participants complained that there needed to be more rules, on household chores, substance use and likewise issues, while others complained that there were too many rules.

“ … . it seemed like there were more rules than in a shelter [… .] with food, you had to be there in time. If you were five minutes late, they wouldn’t serve you food, as if you were a small child” (participant).

“I think you have to be clear in the rules [… .] And if you don’t follow the rules, than we’re done” (participant).

Many participants of JES complained they had too little private space and that there “are always people, you are never alone”, possibly even more so than in a larger program with more opportunities for withdrawing. A majority of the participants we spoke to after they moved to independent housing relished their new privacy. Some stated that they missed the company of participants. The latter were positive on social life within the self-managed programs. Other participants explicitly referred to distrust in their communication with participants. “I know exactly how much I can tell, and how much I can’t tell. Because if you told something in confidence, within ten seconds someone else knew it as well” (participant).

JES was forced by the municipality to become part of a regular organization, to be eligible for welfare funding, which founding participants and social workers loathed. JES was confronted with similar organizational influences as regular programs, such as having to adhere to safety regulations, being financially accountable and handing over some control to the mother organization, although JES appeared to have a high level of discretionary space. As a consequence of the housing shortage in Amsterdam, the municipality issued guidelines as to who could get housing through JES, thereby influencing who could enter JES. Several of the participants stated that they felt powerless and distrustful towards large organizations. “We have no say, you know. [… .]. They [housing corporations, policy advisors] have certain ideas. [… .] They say [… .] we’ll take it into consideration, but in the end, they decide for themselves” (participant).

A final point of mimicry was (perceived) social distance between participants. Many participants did not engage in self-management, beyond joining house meetings. Often only a small group actually managed JES. Some participants and peer workers were happy with the division, stating that “somebody needs to be the boss” (peer worker), a viewpoint that was mostly shared by those in charge, with support of several of those not in charge. Some of those less engaged, were satisfied with not having to manage the program themselves, as long as those who were, did not interfere with their freedom. Others however stated they disengaged because they felt unwelcome, stating that “the bosses of JES” decided everything and were not interested in the opinion of other participants. “I asked the chairman if I could help and he said ‘no’”(participant).

Those managing the program stated they were welcoming to participants, even though they did not come to meetings, did not contribute ideas or were unreliable. Therefore they had to manage the program themselves. New participants joined an existing program, with established rules and norms, which they could not easily change by themselves, limiting a feeling of ownership. “There was already something there, so their beds were made, and that makes it harder to get the process going” (peer worker). Having the opportunity to engage in the management of a program is associated with empowerment, according to our findings and others (Brown, 2012).

The engagement of participants changed over time. The participant who said that he was disappointed because he was not able to engage, later said: “[now] I get the chance to get involved”. Over time the first author has observed multiple instances of an established group of participants moving out and a new group becoming established. Or as one participant states: “I’ve been here for three years, so it’s been good, constructive, supportive, bad. I’ve seen it all”.

### Differences

Although JES and regular programs share several characteristics, two differences distinguish JES from regular programs: a higher degree of (experienced) freedom individually and collectively and more fluidity in structures that do arise. The freedom is symbolized by a key all participants in JES got.

“That I had my own place. That I had my own key. And that I could decide for myself what I did in life, you know, the personal freedom you have [… .] In other shelters, you were tight to schedules and don’t you dare be late, you don’t have that [at JES].” (participant)

Participants had different conceptions of what freedom in self-management meant and to some extent were free to make their own use of the offered freedom, as discussed in the previous sections.

Participants made, enforced and dissolved rules together. Both participants who found rules to harsh or not strict enough, were able to discuss and decide on the rules together. Structures and interaction patterns, such as division of roles, appeared to be relatively fluid. Participants could choose from an array of roles (passive, active, specific tasks, general management, advocacy) and developed and changed their role over time. The roles peer workers had and how they used their lived experience differed, from facilitating self-management to maintaining order. In some periods, the relation between peer workers and participants mimicked the relation between staff and clients in regular programs, both positive (support, advice) and negative (rule enforcing, paternalism). “[The peer worker] wants to do everything. He almost wants to run your life” (participant).

Facilitators (social workers) were hired by participants and had to account for their functioning towards participants and not towards the mother-organization. As a consequence, facilitators experienced a high level of discretionary space in. Almost all facilitators in JES were academically trained social workers, who believed in self-management, more so than most participants and peer workers. Facilitators had no formal say in the management of JES and focused on supporting participants and peer workers individually and collectively with self-management. In practice, facilitators struggled to refrain from reproducing the role of group workers in regular programs, especially if participants and peer workers did not share their vision of collective self-management or if participants got stuck in the afore mentioned fyke (trap) of self-management. When participants and peer workers were struggling with conflicts or were complaining to facilitators, facilitators had to remind themselves, participants and peer workers that they were not in a position to intervene.

“Participants disagree to what extent I as a facilitator should interfere with people who, in the eyes of other participants, do not move forward [… .] I find that difficult [… .] they need to come to me [… .] but if someone keeps on struggling [… .] when is it legitimate to interfere?” (facilitator).

### Structuration

Following from our exploration of the meaning of JES and the similarities and differences between JES and regular programs from the perspectives of stakeholders, our next step was to explore to what extend processes of structuration are described by members of stakeholder groups. Different views on the desirability of structuration were found, as were several structurations of the values of self-management.

Whether JES needed more or less structure was subject to heavy debate among those involved. Structuration is seen as both positive (not reinventing the wheel) and negative (less opportunity for influence for new participants).

“JES needs to move ahead. […] That they are still talking about that cleaning is a problem, groceries are a problem [… .] we have been talking about that for years [… .] Rules that were made in the past, are now being changed. Is that better?” (participant).

“There used to be a group who were there for a long time. And they had their way of self-management. But the moment people start to move out, other people come, comes a whole different, new society, with ever different participants” (participant).

Rules and procedures were constantly discussed and changed, to the dismay of some of the participants and peer workers but to the benefit of those who wanted to have influence on the program. The level of structure fluctuated over time, enabled by the fluid setup of JES. Some peer workers and participants emphasized the support some of the participants needed to get their affairs in order and the importance of rules that allowed participants to focus on their own life. Others preferred less structure, either because they felt that too much structure was a reproduction of regular programs and/or because too much structure limited the possibility for participants to develop new roles and skills.

“I see a lot of people abusing that freedom. [… .] If you don’t want that, than you should intervene hard, but we don’t want that, because we like to keep our freedom” (participant).

Participants, peer workers and facilitators referred to an unpredictable and unique trajectory that each participants goes through, “*which can’t be steered”* (peer worker), although they also mentioned examples of encouragement and stimulation, for instance by processes of social comparison with and social learning from other participants who are making progress.

We found several examples of how self-management was embodied in the structure, including a key all participants had and a set Monday meeting with actual influence on all aspects of JES, from household to hiring and redecorating. Rituals within JES were far less codified compared to for instance self-help groups (Brown, Tang, & Hollman, 2014). The structure of self-management appeared to allow attuning to the wishes and needs of participants. Participants were able to work on their own problems in their own way and in their own time.

## Discussion

In this paper we explored the meaning of self-managed shelters compared to regular institutional shelters from multiple stakeholder perspectives. We have analysed JES, a self-managed institutional homeless programs, as an institution, which is given little to no attention in literature on self-organized care (Brown, 2012). Our analysis provided new insights into self-management and into how JES was similar to and different from regular institutional care, which also offered new insights into regular institutional care. For our analysis we build on Goffman’s concept of a *total institution* (1961) and institutional theory, describing how an institutional setting influences behaviour and how behaviour in turn influences an institutional setting.

The main similarities between JES and regular homeless care follow from the institutional character of JES, although this was not expected. Participants in JES lived together with people whom they did not choose themselves and with whom they mainly shared a background in homelessness. Having to share space and facilities (kitchen, bathroom), in combination with a vulnerable background that participants shared, caused various tensions, such as an ongoing discussion on rules and enforcement of rules and participants withdrawing from social processes. The description of communication that was given appears to echo the description of strategic interaction, focused on distrust and power, that others described in regular programs (Goffman, 1961; Hanrath, 2013). In periods, there was increasing social distance between those more and less engaged. JES is also part of a larger organization, financed by the municipality and dependent on the municipality and housing organizations for access to independent housing, although JES appeared to have a high level of discretionary space. A major difference between JES and regular programs was the personal and collective freedom of JES participants, symbolized through a key all participants had. Another difference was fluidity in the structure and interaction patterns in JES, which allowed roles and positions to develop and new participants to re-discus rules they felt were not suited.

Compared to Goffman’s (1961) description of institutional care, participants in JES were much less confronted with processes of mortification, although some participants did behave similar with mortification, e.g., passive and focused on rules, possibly because of learned helplessness in previous regular programs. Influencing learned helplessness of homeless people is a complex effort (Van Regenmortel, Demeyer, Vandenbempt, & Van Damme., 2006). Other institutional processes were reproduced in JES as well, which can be partly explained by similarities in the setup. Changing the management structure of the program to self-management can create more individual and collective freedom and fluidity, although it is not enough to diminish institutionalization, in line with the theoretical argument that individuals reproduce and strengthen institutions themselves (Giddens, 1984). Further research is needed to better understand the role of social workers and peer workers.

From a critical perspective, it could be argued that processes of social control appeared internalized by participants and peer workers, possibly because participants have internalized the need for control from regular programs. Developing structures appeared to be stimulated to some extent by external pressures and an unnatural setting of strangers living together based on a shared vulnerability. JES promotes moderate identity transformation of participants, like regular programs, albeit towards empowerment. Some participants experience identity transformation as positive, while others echo state that identity transformation is unwanted social control. Others respondents and authors argue that empowerment is an acceptable balance between freedom of choice and offering support (Rappaport, 1981). Overall, most participants prefer JES over regular homeless care, even those who are critical of the institutional aspects of JES as discussed in this paper.

### Strengths and limitations

Our data is predominantly narrative, requiring reflective and verbal capacities, which risks underrepresentation of less verbal respondents (Bryman, 2008). Participants who were less enthusiastic about JES are underrepresented in our data, because of limitations in recruitment. In our analysis and our presentation of the data we emphasized an authentic representation, especially of less verbal and/or critical participants, to counter underrepresentation, and to explore alternative and competing explanations (Abma et al., 2009). Non-verbal or more structured (less reflexive) data gathering in future research can triangulate our findings (Bryman, 2008). We did not use data specifically collected with a focus on institutional factors, nor did we collect data specifically focused on interaction patterns, future research focused specifically on the issues described in this paper might provide furthers insights. A strength of our research is the first author’s prolonged engagement with JES, combined with peer debriefings, which enabled him to observe developments over time, gain insight into the dynamics of self-management and increase the representability of observations (Bryman, 2008). Engagement of participants, peer workers and social workers in the research increased the authenticity of our findings (Lincoln & Guba, 1985). In future work we will reflect more on our methodology and the role of researchers and participants in facilitating learning through our research.

### Practical implications

JES started with an ambition to have very little structure. Over the years, it has developed some structures that are beneficial (regular meeting, clear decision structure) and fluctuated with other structures, supporting the argument that participants do influence institutionalization (DiMaggio & Powell, 1983; Giddens, 1984) and that structures can be enabling as well (Adler & Borys, 1996). Our research also offers some nuance to critics of staff in regular institutional programs, who are only a part of the actors and influences in processes of institutionalization, since several aspects of institutionalization appeared tied to an institutional context, rather than to staff. JES revealed opportunities for introducing freedom of choice and fluidity in structure, rules and roles within an institutional setting, facilitating unique individual processes of empowerment.
